# Impact of Air Pollutants on Lightning Activities in Kathmandu, Nepal

**DOI:** 10.1155/tswj/8125403

**Published:** 2026-03-19

**Authors:** P. B. Adhikari, J. S. Dhami, K. N. Poudyal, N. P. Chapagain

**Affiliations:** ^1^ Department of Physics, Tri-Chandra Multiple Campus, Tribhuvan University (TU), Kathmandu, Nepal, ioe.edu.np; ^2^ Department of Applied Sciences and Chemical Engineering, IOE Pulchowk Campus, TU, Lalitpur, Nepal; ^3^ Department of Physics, Amrit Campus, TU, Kathmandu, Nepal

**Keywords:** atmospheric pollutants, lightning, relative humidity, temperature

## Abstract

An electric field mill (EFM‐100) short‐range sensor has been installed on the rooftop of the six story building of Kathmandu BernHardt College, Tribhuvan University (TU), Bafal, Kathmandu for continuous monitoring of lightning events within a 38 km in aerial distance. It constantly monitors lightning activities and activates graphical display alerts on a connected computer. The EFM‐100 monitor software displays a real time graph of the atmospheric electrical field, as well as the distance of lightning strikes within the specified range. Lightning strikes were recorded over a 3‐month period, spanning 57 days, primarily during the both premonsoon and monsoon seasons, from May 19 to August 18, 2024. In addition, the effect of lightning events on atmospheric pollutants such as PM_1_, PM_2.5_, PM_10_, relative humidity, temperature, and particle density of ozone per cubic centimeter were analyzed. These atmospheric parameters were obtained from the Central Department of Environment Science, TU, Nepal. The results clearly show that the atmospheric pollutants PM_1_, PM_2.5_, and PM_10_, found to be higher during the lightning events. PM_2.5_ concentrations increased by an average of approximately 18%, PM_10_ by about 12%, and ozone by about 22% compared with prelightning background levels. Similarly, the atmospheric temperature increased by 1.5°C–2.0°C during the strong lightning events followed by a gradual decrease thereafter. These findings provide clear evidence of the magnitude of pollutants and thermal changes associated with lightning. It was also observed that relative humidity is inversely proportional to temperature, as it rises due to lightning phenomena. Furthermore, lightning activity was found to significantly increase with rising concentrations of ozone particles in the atmosphere.

## 1. Introduction

Lightning is a significant safety hazard that can lead to injuries or even fatalities. It is a natural phenomenon involving a discharge process. While lightning is often considered dangerous, it can also have beneficial effects on both humans and the environment. In 1752, Benjamin Franklin conducted experiments that helped establish lightning as a scientific natural phenomenon, making it easier to study and manage safely.and Cooper Holle [1] reported that lightning is the main disaster that affects all parts of the world; among them, tropical and subtropical areas are affected more. Adhikari [2, 3], reported that the ratio of injured people to the death people is 3:1 due to lightning during the period of 2011–2021 in Nepal. In the Himalayan region, Adhikari [4] found that 62.8% is cloud to cloud, 20.4% is cloud to ground lightning, 9.5% are unusual events, so implementing clear safety protocols is essential to protecting employees during thunderstorms. Additionally, lightning plays a crucial role in the Earth′s ecosystem, contributing to both the origin and survival of life. One notable effect is the formation of hydrogen cyanide (HCN) at extremely high temperatures, which can occur during lightning strikes and is important for life on Earth [5]. Lightning is essential for the environment and ecosystem, playing a crucial role in natural processes such as nitrogen fixation and atmospheric chemistry. To detect lightning, various methods and technologies are used, including ground‐based sensors, satellite monitoring, and electromagnetic wave detection. These systems help track lightning activity, improve weather forecasting, and enhance safety measures.

The COVID‐19 pandemic provided a unique opportunity to observe these interactions, acting as a natural experiment to study the direct impact of reduced anthropogenic emissions on atmospheric processes [6]. They demonstrated a significant decrease in premonsoon lightning activities, with a reduction of 49.16% compared with previous years (2010–2019) in megacities like Kolkata, India [6, 7]. This sharp decline in lightning was strongly correlated with substantial reductions in major air pollutants: PM_10_, NO_2_, SO_2_, O_3_, and aerosol concentration, which decreased in the range of 40%–68% with respect to prelockdown levels. Additionally, air quality improvement during lockdowns was associated with notable reductions in urban temperatures, with Kolkata experiencing a 2.5°C drop [8, 9]. Similarly, polluted urban environments investigating the impact of air pollutants on lightning activities in Kathmandu, Nepal, are critically important. We have studied the influence of lightning characteristics and contributed to urban atmospheric impacts on Kathmandu city in the Himalayan region.

Geographically, Nepal is unique and complex terrain. Its altitude varies from 58.0 to 8848.86 m [3], so different types of climates are found. There are many more lightning hazards and storms that occur every year during the premonsoon and monsoon seasons. Nepal is an agricultural country where more than 66% of people are farmers and they live in rural and remote areas. Every year many more farmers and workers are affected by the lightning either in farm places or other working activities outside their houses. Besides this, most of the rural farmers are not aware of the safety rules and regulations about lightning and storms ([10, 11]. Aerosols, relative humidity (RH), rainfall, and temperature play a vital role in generating the lightning or thunderstorms in the atmosphere. However, the role of aerosols in thunderstorm electrification is still not fully understood. Research indicates that thunderstorms developing in smoke‐laden air masses generate a high amount of positive polarity lightning (+CGs) [12]. Jnanesh et al. [13] showed that the nonlinear relation between aerosol and lightning and atmospheric humidity is the main factor to decide their relation. According to Murray et al. [14], the occurrence of positive flashes increases due to the impact of aerosols from forest fires. Orville et al. [15] suggested that flash densities rise with increased air pollution from human activities. Additionally, drier climates might result in more suspended aerosols, dust, and CNN, which could impact cloud microphysics and electrification processes [16].

Rapid urbanization and industrialization have globally intensified atmospheric air pollution, leading to significant environmental and health challenges, particularly in densely populated urban centers [8]. In major metropolitan areas, including several in India like Kolkata, which frequently experience high concentrations of PM_2.5_, PM_10_, NO_2_, SO_2_, CO, O_3_, and aerosols, predominantly from anthropogenic sources such as vehicular emissions, industrial activities, and waste burning. Beyond the severe health impacts, which include millions of premature deaths globally due to PM_2.5_ exposure, these pollutants profoundly influence local and regional meteorological phenomena. A growing body of research indicates a complex yet strong relationship between urban air pollutants and lightning activity [17–20].

Lightning is the spark of electricity due to the opposite charge of the thunderstorm. Lightning requires heavy uplift, whereas precipitation may occur with moderate updrafts [21]. It is one of the deadliest meteorological phenomena and causes damage to objects and fire forests [1, 22, 23]. A noticeable increase in CG lightning density during seasonal cycles under high aerosol optical death (AOD) conditions across all regions. However, both CG lightning density and total AOD showed declining trends in their interannual variations across the three study regions over the study period [24].

Furthermore, remote sensing (RS) technologies are indispensable for comprehensively monitoring and analyzing environmental changes, particularly in dynamic urban areas and for large geographical coverage where ground‐based data are often insufficient ([25, 26] Advanced satellite systems offer diverse data for assessing air quality, land use/land cover (LU/LC) changes, and atmospheric phenomena. Planet′s SkySat and PlanetScope constellations provide daily datasets. PlanetScope data offers RGB, multispectral, and near‐infrared (NIR) bands at a 3.7 m spatial resolution, with four bands and 3 m pixel sampling, delivering 8‐bit visual and 16‐bit analytic radiometric resolution in GeoTIFF format. SkySat provides higher‐resolution data (0.5 m) in RGB, NIR, and panchromatic (PAN) bands, with 5 bands and 0.5 m pixel sampling, offering visual, panchromatic, and pan‐sharpened multispectral products. Both systems achieve positional accuracy of less than 10 m RMSE and provide various products, including global base maps and historical archives. Furthermore, MODIS (Moderate Resolution Imaging Spectroradiometer) Terra and Aqua satellites, provided by NASA, are widely utilized for measuring aerosol concentrations, offering daily AOD at 10:30 and 13:30 local time, crucial for correlating with PM_2.5_ levels [6]. For lightning activity, the Lightning Imaging Sensor (LIS) aboard the Tropical Rainfall Measuring Mission (TRMM) satellite provides valuable data on cloud‐to‐ground and intercloud lightning by detecting illumination pulses [6].

Similarly, the vast amounts of data collected by RS systems are processed using various image classification techniques to categorize pixels into meaningful classes [27]. Common supervised classification algorithms include parallelepiped classification (PPC), minimum distance classification (MDC), Mahalanobis distance classification (MaDC), maximum likelihood classification (MLC), spectral angle mapper classification (SAMC), and spectral information divergence classification (SIDC). These techniques find broad applications in areas such as soil moisture mapping, urban growth estimation, river and canal mapping, and particularly in change estimation. The reliability of classification results is rigorously assessed through metrics like user accuracy (Ua), producer accuracy (Pa), omission error (Oe), commission error (Ce), kappa coefficient (Kp), and overall accuracy (Oa). Studies consistently show that MDC, MaDC, and MLC algorithms generally yield the most reliable results and highest classification accuracies and kappa values. Specifically, MLC has been found to produce the highest classification accuracies in most cases due to its sophisticated probabilistic procedure for assigning pixels to specific categories. These sophisticated RS tools and analytical methods are therefore essential for precisely mapping, monitoring, and understanding the intricate relationship between air pollutants and atmospheric phenomena like lightning [25, 26].

However, the interaction between aerosols and lightning is complex. The aerosol effect is mainly regulated by humidity. In general, the correlation between aerosol and lightning for the dry cases is the opposite to that of the wet cases. CAPE and surface temperature could also affect the aerosol effect over lightning. At low aerosol levels, aerosols delay precipitation, which enhances the electrification process and increases lightning. However, at high aerosol levels, aerosols can absorb solar radiation, warming the aerosol layer and cooling the surface, which stabilizes the lower atmosphere and decreases lightning activity [28]. Shi et al. [29] found that aerosols can affect lightning by altering cloud microphysics, with a positive correlation between lightning flash rate and AOD under relatively clean conditions (AOD < 1.0). This correlation weakens at higher aerosol concentrations (AOD > 1.0), likely due to conflicting effects of aerosol radiation and microphysics.

To better understand the effects of particulate matter (PM) on Earth′s climate and human health, it is essential to monitor PM_2.5_ globally, despite the challenges presented by the variability of these submicron aerosols. Thornton et al. [30] proposed that shipping emissions alter storm cloud microphysics, doubling lightning activity over major shipping lanes in the Indian Ocean and South China Sea. Added aerosols can cool the sea surface and reduce CAPE, weakening thermodynamic effects on lightning. Nonetheless, stroke densities (mean, IC, CG, + IC, and + CG) increase significantly compared with nearby cleaner areas [31]. Lightning, an electrostatic discharge resulting from charge separation within clouds, is influenced by the microphysical processes of cloud formation and electrification, which are significantly altered by atmospheric aerosols and other pollutants acting as cloud condensation nuclei (CCN) [18–20]. High concentrations of pollutants like PM_10_, NO_2_, SO_2_, and O_3_ have been consistently linked to increased cloud‐to‐ground and inter‐cloud lightning flashes, as they modify cloud droplet sizes, affect precipitation processes, and enhance cloud water content, thereby impacting the formation of ice particles crucial for thunderstorm electrification [18–20]. The strengthened maritime convective clouds, stimulated by added aerosols, lead to more frequent and intense mixed‐phase development that favors lightning [31]. The relationship between lightning activity and aerosols differs based on aerosol loading. Thornton et al. [30] explain that aerosols, a key element of air pollution, significantly affect lightning activity by interacting with clouds and being influenced by human activities. Thus, studying their impact on lightning and climate variables across various regions is essential. Khain et al. [32]; Wang et al. [33]; Li et al. [34] studied and explored the relationship between lightning occurrence and air pollution. Atmospheric pollutants impact cloud systems directly by modifying the radiation balance and indirectly by affecting cloud dynamics and microphysical characteristics. In polluted areas, aerosols can enhance the number of CCN and cloud droplets, which are carried upward by updrafts and transformed into supercooled liquid, possibly initiating lightning [35]. Aerosol effects are classified into radiative and microphysical processes [36] and are vital in driving convective activity, largely dependent on atmospheric humidity [37].

Lal et al. [37, 38] also suggested that atmospheric humidity is a key factor in controlling the effects of aerosols on both clouds and lightning. The interaction between aerosols and atmospheric moisture influences cloud droplet size. When humidity remains constant, an increase in aerosol concentration leads to smaller cloud droplets [39]. These smaller droplets can ascend to higher altitudes, enhancing ice‐based electrification processes that may trigger lightning [16, 37, 38, 40]. Furthermore, a correlation has been noted where larger cloud droplets are linked to high humidity and low AOD, reinforcing the connection between aerosols, humidity, and cloud droplet size. Consequently, the microphysical effects of aerosols play a vital role in influencing lightning activity. Altaratz et al. [28] found that increased concentrations of Amazonian smoke particles initially resulted in deeper clouds and more frequent lightning up to a moderate level of aerosol loading. However, beyond this threshold, further rises in aerosol concentration led to a decline in lightning activity, forming a “Boomerang”‐shaped relationship between flash density and AOD. The “Boomerang” relationship between flash density and aerosols may be attributed to both the microphysical and radiative effects of aerosols. By absorbing and scattering solar radiation, aerosols reduce the amount of sunlight reaching the Earth′s surface.

Cleaner air has the opposite effect on cloud electrification, leading to reduced lightning activity because water is concentrated in fewer CCN and cloud droplets [41]. In less polluted environments, rainout occurs earlier, and vertical transport into the mixed‐phase region is weaker, resulting in lower lightning frequency [35]. Research suggests that convective processes enhancing cloud electrification increase up to a certain PM threshold, after which they decline, as higher particulate concentrations block sunlight and stabilize the atmosphere [35, 42–44]. Pinto Neto et al. [45] identified a nonlinear relationship between lightning activity and aerosol concentrations during the COVID‐19 shutdown, a time characterized by a notable decline in air pollution. Similarly, Perez‐Invernon et al. [46] analyzed pollutant levels and lightning activity in northern Italy before and after the lockdown, concluding that 60% of the decrease in lightning occurrences was driven by meteorological factors, while the remaining 40% resulted from reduced aerosol emissions.

The lightning and its effects are still not understood. In the context of Nepal, there is more positive lightning, which is strange for the researchers. The role of aerosols in thunderstorm electrification is also interesting at this complex terrain in the Himalaya region for the researcher. Even if it is still not fully understood, it may relate to doing more research about the lightning thunderstorm and its effects on the atmospheric parameters. The main objective of this research is to determine the relation of the impact on lightning due to air pollutants.

## 2. Methods and Instrumentations

### 2.1. EFM‐100 Short‐Range Sensor

**Figure 1:**
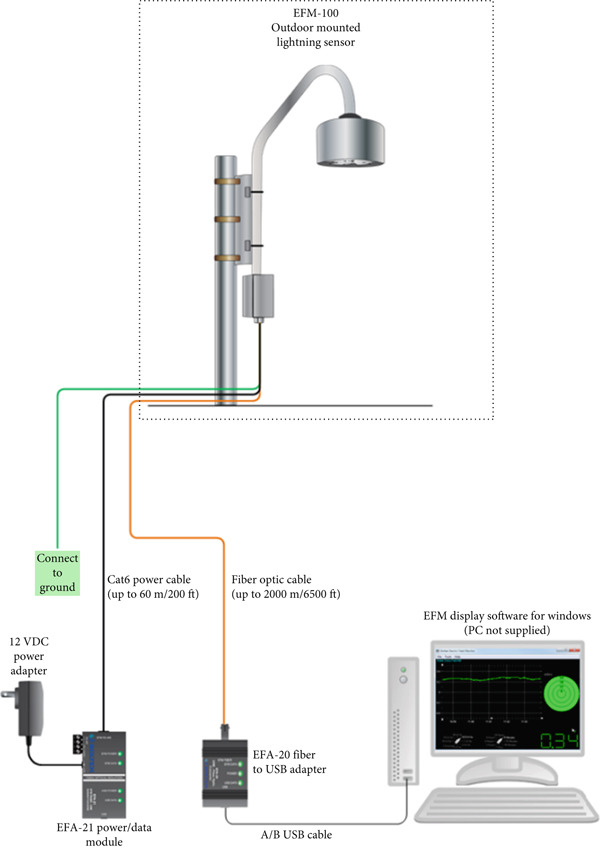
EFM‐100 fiber optic connection diagram.

EFM‐100 short‐range sensor has been installed on the rooftop of the six‐story building of Kathmandu BernHardt College, Tribhuvan University (TU), Bafal, Kathmandu for continuous monitoring of lightning events. Bafal was chosen as the study site because it is located in the central urban core of Kathmandu Valley, which experiences both high anthropogenic emissions and frequent thunderstorm activity. Additionally, an air‐quality monitoring station is nearby this station from which the data were taken for the study. The EFM‐100C atmospheric electric field monitor is a robust instrument designed to measure atmospheric electric fields with high precision. Equipped with advanced hardware and software, it operates within an electric field range of −20 kV/m to +20 kV/m, with a typical range of interest from −5 kV/m to +5 kV/m. Its rapid response time of 0.1 s and digital output resolution of 0.01 kV/m ensure accurate and timely measurements. Data transmission is facilitated by a 50/125 multimode fiber optic cable with ST connectors, formatted using RS232 protocol at 9600 baud, with 8 data bits, no parity, and 1 stop bit. The device can be powered by 12–15 VDC or standard AC supplies (120/220 VAC) and is equipped with a durable, ball‐bearing brushless DC motor mounted on a 3/4" NPT threaded pipe. The compact design, measuring 6.7 in. in diameter and 5 in. in height, weighs only 2.2 lbs (1 kg), making it portable and easy to install (as shown in Figure 1). Its robust construction ensures reliable performance under extreme temperatures ranging from −40°F to +140°F −40°C to +60°C).

The EFM‐100C Monitor operates on the principle of periodic exposure and shielding of sensing electrodes to measure atmospheric electric fields. The device uses a motorized rotor to alternately expose and shield the electrodes from the electric field, creating a dynamic flow of charge. When the electrodes are exposed, an electric charge is drawn from the ground through a sense resistor, and when shielded, the charge flows back to the ground. This movement of charge generates an alternating current (AC) voltage across the sense resistor, proportional to the strength of the electric field. The generated voltage is amplified and processed to produce a digital or analog output. The relationship between the electric field (E) and the measured voltage (V) is calibrated to ensure precise readings. This principle allows the device to detect variations in the electric field caused by environmental phenomena, such as thunderstorms or lightning strikes.

**Figure 2:**
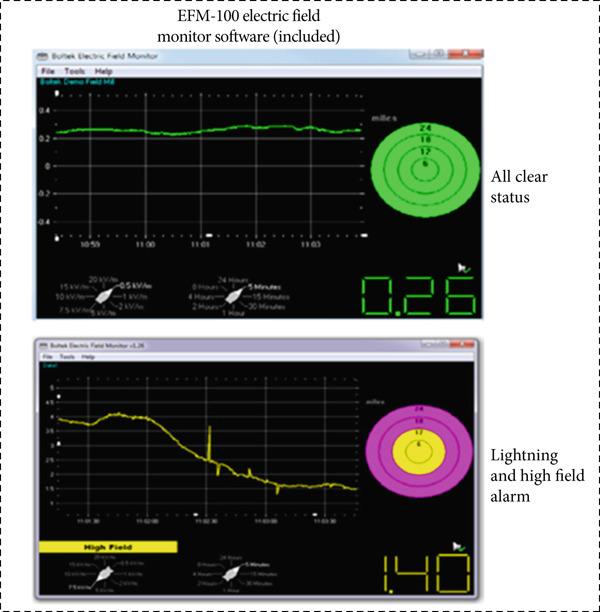
Monitoring of lightning data in our research laboratory.

The EFM‐100C is designed to monitor electric fields and detect lightning within a range of 38 km. Its customizable high‐field alarms and lightning distance ranges allow for tailored operation. The software displays real‐time graphs of atmospheric electric fields and lightning strike distances, providing essential data for monitoring environmental changes. When lightning is detected, the software activates graphical alerts, resetting to a green “all‐clear” state when no lightning is detected within the configured time and the electric field levels return to safe thresholds (as shown in Figure 2). The software supports multiple Microsoft Windows operating systems, offering features such as high‐field alarms, very high‐field alarms, and customizable lightning detection settings. The history graph allows users to analyze trends with adjustable time ranges from 5 min to 24 h, along with vertical scales up to 20 kV/m.

### 2.2. Setup of the Experiments

**Figure 3:**
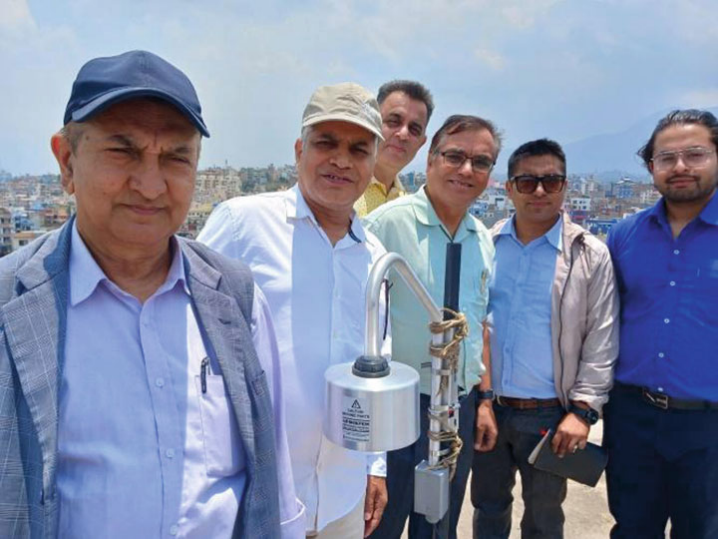
Snap shots taken during the installation of the instrument in our research laboratory in Kathmandu.

The EFM‐100 is best suited for installations when it is placed 2–3 feet above the roof of the building. With the inverted kit as shown in Figure 1, it comes with a tripod for quick and easy setup as well as an inverted mounting kit to greatly reduce the number of false strikes during precipitation. Lightning and high field alarm active EFM‐100 monitor software have a high accuracy 38 km range for local activity. For far away lightning it is shown by yellow color, whereas for the high field it is represented by red color. The clear display is represented by green color and hence it gives distances of the lightning occurring, its durations, and high electric field levels. Some photos are given below in Figure 3 that were taken during the installation of the instrument in our research laboratory.

In addition, real‐time pollutant data such as PM_1,_ PM_2.5_, PM_10_, ozone, RH, temperature were obtained from air‐quality monitoring station at the Central Department of Environmental Science, TU. The nearby location of these two instruments ensured consistency, minimal spatial bias, and reliable comparison between lightning activity and pollutant concentration. Similarly, observed lighting data were time‐stamped and aggregated into 1‐min averages for consistency, and pollutant data from the nearby station are also archived as 1‐min averages. Synchronization was achieved by aligning both datasets according to their UTC and NPT‐based time stamps. This approach allowed us to compare pollutant concentration during prelightning, lightning, and postlightning intervals within the same temporal framework.

## 3. Results and Discussion

This study examines the results of lightning observations and their impact on atmospheric air quality parameters, including PM_1_, PM_2.5_, PM_10_, RH, temperature, and ozone concentration. The analysis also takes cloud conditions into account during lightning events. While lightning tends to occur at specific times of the day, its influence on air quality persists throughout the day, both before and after the event. After installing the instrument, lightning data were recorded over the first 3 months. During this period, lightning was detected on 57 separate days, as summarized in Table 1. From the observed data, we have selected lightning events from 2 days (May 19 and June 5, 2024) during the premonsoon period, while another 2 days (July 6 and 16, 2024) during the monsoon period for detailed analysis. These selected lightning events are highlighted in Figures 4, 5, 6, and 7. Figures 4 and 5 illustrate lightning activity alongside atmospheric parameters during the premonsoon period, whereas Figures 6 and 7 highlight similar data for the monsoon season. The observed lightning events occurred at distances ranging from 3 km to 38 km from the measurement station in Kathmandu, encompassing both the premonsoon and monsoon seasons of 2024. The frequency and intensity of lightning varied across different days, as outlined in Table 1. Examining these variations helps in understanding the role of lightning in influencing air quality over time.

**Table 1:** The number of lightning events that occurred on various days and air quality parameters on the corresponding days.

**S.N**	**Date of lightning**	**No. of lightning events**	**Air quality parameter (maximum value)**					
			**PM**_**1**_ ** (*μ*g/m** ^**3**^ **)**	**PM**_**2.5**_ **(*μ*g/m** ^**3**^ **)**	**PM**_**10**_ **(*μ*g/m** ^**3**^ **)**	**Relative humidity (%)**	**Temperature (°C)**	**Particle density (Um003)** **(particles/cm** ^**3**^ **)**
1.	2024‐05‐19	3	33.68889	67.79894	55.56958	51.2342265	35.36091	8469.517
2.	2024‐06‐04	5	35.92643	59.77837	70.35625	48.0520830	32.77095	9258.233
3.	2024‐06‐05	7	32.09268	53.48491	63.31275	52.9717261	33.50183	7615.154
4.	2024‐06‐06	11	29.94116	49.16457	60.80683	56.6180058	35.99435	6917.772
5.	2024‐06‐07	6	27.63532	44.12818	52.73036	49.9809027	34.93139	5904.782
6.	2024‐06‐08	11	101.7077	189.2563	205.3247	54.1784099	37.82955	25009.89
7.	2024‐06‐09	2	36.32857	60.23551	70.68639	49.9827881	37.48809	9306.54
8.	2024‐06‐10	4	40.11314	68.64867	78.84186	46.6886412	38.64504	10166.67
9.	2024‐06‐11	21	39.13254	65.78591	75.37877	46.3902781	36.16666	10082.64
10.	2024‐06‐12	4	37.22374	65.6249	74.85144	49.0127973	39.67979	9767.288
11.	2024‐06‐13	9	39.17827	68.6995	76.05392	48.8221728	36.71289	10209.55
12.	2024‐06‐14	20	40.06537	71.44444	78.92779	51.6622519	36.35730	10413.94
13.	2024‐06‐15	12	37.46515	66.59113	75.11797	52.3633731	38.41349	9737.113
14.	2024‐06‐16	50	32.1619	52.63119	65.24811	52.3486108	34.58334	7739.419
15.	2024‐06‐17	11	31.34083	49.58066	61.27247	61.9163262	35.52688	7458.124
16.	2024‐06‐18	22	38.24405	64.67952	73.73095	59.0086609	33.85792	9836.819
17.	2024‐06‐19	29	30.9813	50.8121	62.42029	56.6262987	35.12768	7301.197
18.	2024‐06‐20	2	27.79065	44.48946	53.66284	59.9135517	34.99929	6062.75
19.	2024‐06‐21	6	21.93146	34.48292	39.35125	50.0946429	33.69175	4342.85
20.	2024‐06‐22	22	27.53145	44.49871	52.31066	53.9260915	37.06195	6046.312
21.	2024‐06‐25	29	29.71333	48.6955	59.81133	51.8051945	34.19140	6829.588
22.	2024‐06‐26	20	26.64529	45.89425	56.41007	55.9313488	30.26548	5708.932
23.	2024‐06‐27	3	28.08854	45.4372	55.49702	56.2739578	33.74929	6265.199
24.	2024‐06‐28	17	34.42556	59.32333	63.00333	63.1563978	34.76667	8414.222
25.	2024‐06‐29	27	19.33592	30.90025	33.85763	58.9291667	30.87421	3666.736
26.	2024‐06‐30	10	24.83973	40.74231	48.62925	59.1642853	34.22917	5137.864
27.	2024‐07‐02	18	28.25	42.25	49.65	79.2333374	29.00000	6440.5
28.	2024‐07‐03	7	24.79225	38.38265	44.81378	58.8666664	34.05861	5049.494
29.	2024‐07‐04	6	21.8506	33.9998	38.41369	61.2642860	31.83052	4294.746
30.	2024‐07‐05	17	26.10298	41.07966	48.97262	58.2592862	32.97009	5491.382
31.	2024‐07‐06	26	29.75255	46.50544	55.55238	66.8297077	25.05001	6821.143
32.	2024‐07‐07	18	27.07143	41.65714	49.77143	62.2952375	28.58572	5734.571
33.	2024‐07‐08	6	22.16667	36.40833	42.75833	65.5913689	33.20215	4402.917
34.	2024‐07‐09	7	24.3375	40.91428	48.35893	59.1811218	32.31289	4959.643
35.	2024‐07‐10	21	26.97103	46.02679	55.85813	61.7505954	30.88056	5911.619
36.	2024‐07‐21	9	22.475	34.18333	38.2	61.2452778	28.85967	4400.167
37.	2024‐07‐22	3	25.91151	40.04008	47.52976	62.1451981	32.50049	5455.115
38.	2024‐07‐23	26	21.332	31.49533	34.94133	62.322024	33.65609	4314.423
39.	2024‐07‐24	28	65.02401	139.7384	135.476	62.28438	31.20417	16387.18
40.	2024‐07‐25	15	20.09905	29.45658	31.26623	62.4543155	34.54532	3786.725
41.	2024‐07‐26	7	15.50298	22.64375	23.40476	61.1337795	36.64217	2893.548
42.	2024‐07‐27	3	22.7872	34.16463	38.52887	59.7253248	33.52499	4524.127
43.	2024‐07‐29	2	22.11365	33.62655	37.76639	61.95655	37.48333	4470.838
44.	2024‐07‐31	9	23.31518	34.09643	38.29732	62.55353	32.71825	4692.72
45.	2024‐08‐01	27	19.675	28.64502	31.40032	56.9252	36.40203	3996.575
46.	2024‐08‐02	1	20.17341	30.57163	33.44345	59.32738	35.50045	3917.754
47.	2024‐08‐06	16	19.01342	28.39632	31.7026	62.87619	31.26443	3936.478
48.	2024‐08‐07	9	20.70729	32.67758	36.25888	60.24048	32.88948	4145.699
49.	2024‐08‐08	5	23.07158	35.43199	40.44588	61.38948	34.41518	4603.122
50.	2024‐08‐09	7	31.13115	49.09504	54.03472	62.7189	32.57991	6942.601
51.	2024‐08‐11	8	22.93426	36.83545	43.11984	64.23408	31.60466	4605.16
52.	2024‐08‐12	3	24.80471	39.37569	46.61062	59.26288	34.24845	5205.779
53.	2024‐08‐13	13	26.11815	40.90402	49.01136	63.02485	35.16503	5619.108
54.	2024‐08‐14	8	23.79172	37.29826	43.6943	63.39077	34.02039	4825.676
55.	2024‐08‐15	26	14.83021	22.88021	23.98705	59.60982	36.09792	2745.238
56.	2024‐08‐16	16	18.49	29.27167	31.65	70.45789	36.23616	3546.8
57.	2024‐08‐17	3	20.24241	29.79018	33.1128	61.08309	33.16806	4175.908

Figure 4(a) Lightning events representing peak values of intensity on May 19, 2024 and (b) diurnal variations of various air quality parameters such as intensity, PM_2.5_, PM_10_, PM_1_, relative humidity (RH), atmospheric temperature (T), and particle density on the corresponding day (May 19).

**Figure:**
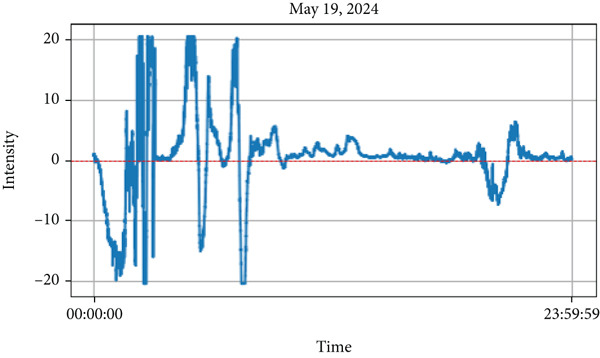
(a)

**Figure:**
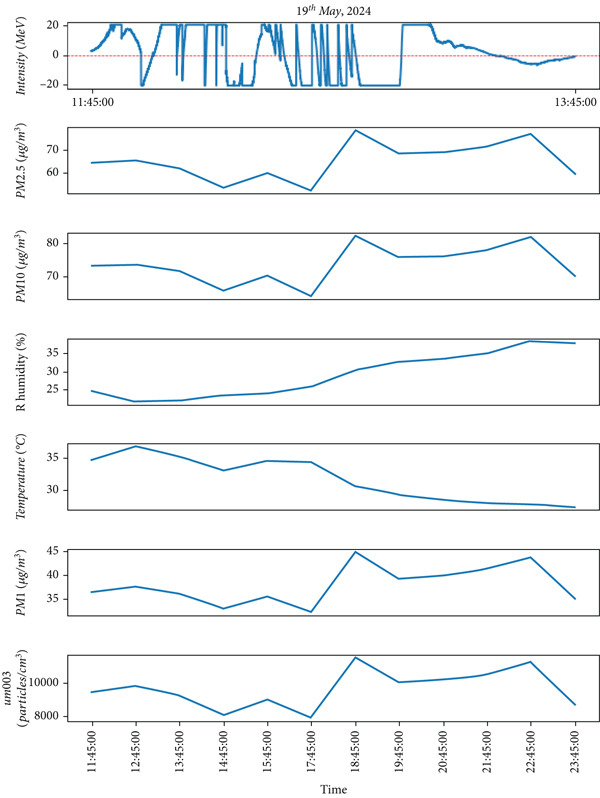
(b)

Figure 5Lightning events (within 30 min) representing peak values of intensity on June 5, 2024 in the upper and diurnal variations of various air quality parameters such as intensity, PM_2.5_, PM_10_, PM_1_, relative humidity (RH), atmospheric temperature (T), and particle density on the corresponding day is given in lower.

**Figure:**
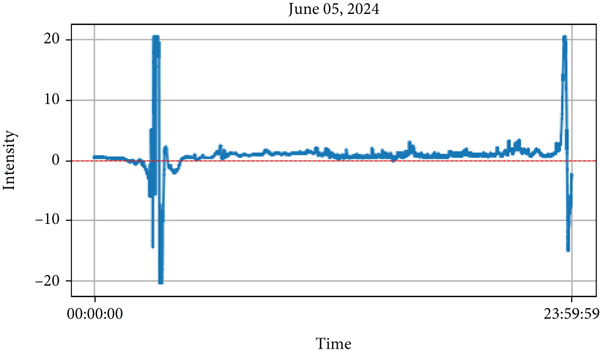
(a)

**Figure:**
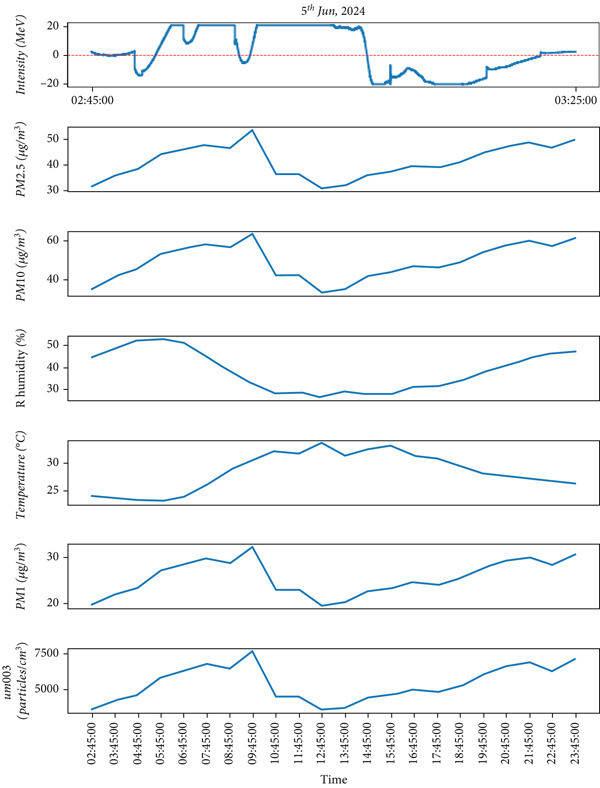
(b)

Figure 6The different parameters of the atmosphere with lightning on July 6, 2024, during the monsoon period. The lightning phenomena occurred in the upper part, and the lower part represents atmospheric parameters in which the top most represents lightning and the remaining atmospheric parameters.

**Figure:**
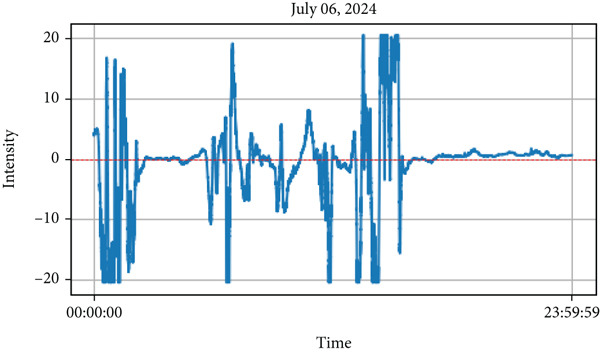
(a)

**Figure:**
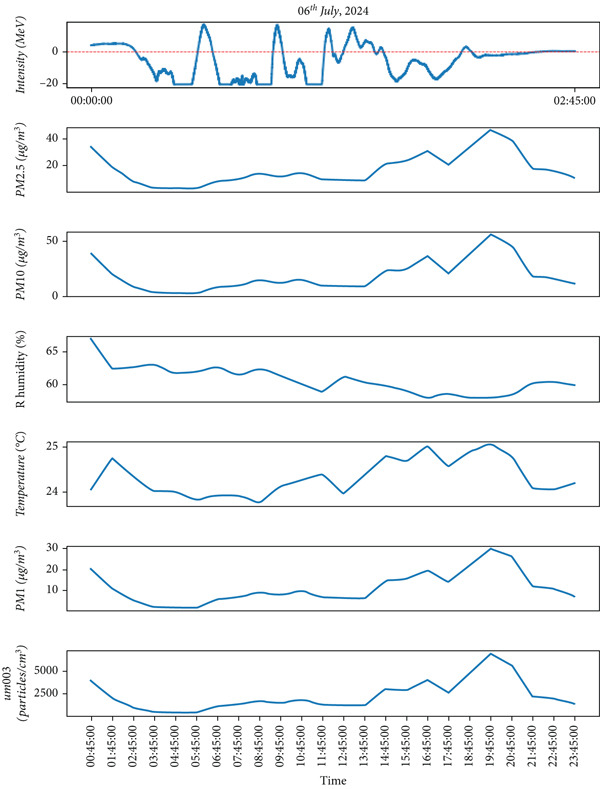
(b)

Figure 7The different parameters of atmosphere with lightning on July 16, 2024 during the monsoon period. The figure (a) the lightning phenomena occurred and (b) represents atmospheric parameters in which the top most represents lightning about 30 min.

**Figure:**
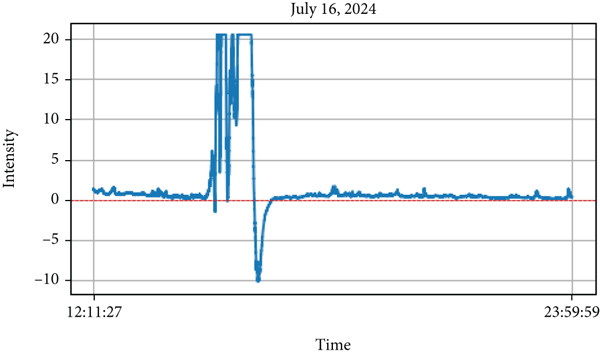
(a)

**Figure:**
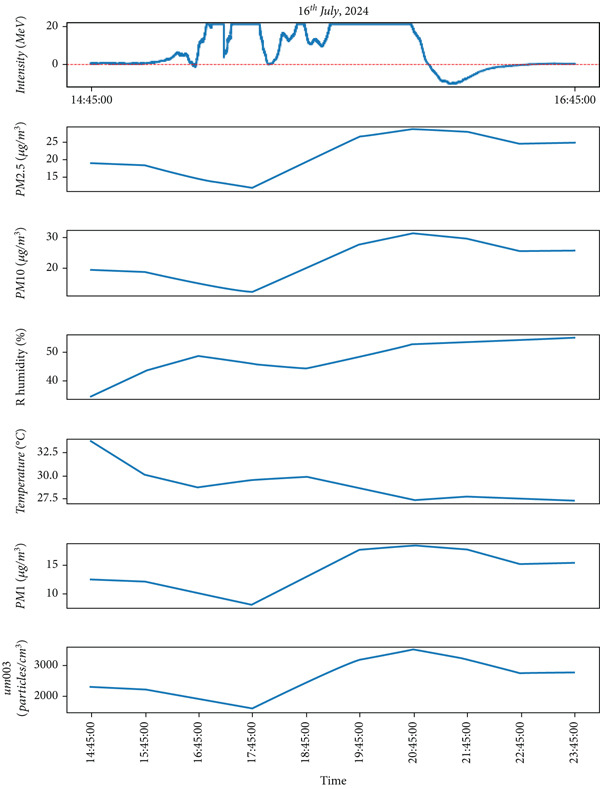
(b)

We have limited data to analyze the occurrence of lightning events for premonsoon season (March–May) as the instrument was deployed on May 18, 2024. However, lightning events were recorded frequently during the monsoon period. From Table 1, lightning occurred during the beginning of the monsoon very frequently, up to 50 lightning strikes (e.g. on June 16, 2024). However, from a case study of lighting on May 19, 2024, the premonsoon season, the relationship between lightning intensity and various air quality parameters PM_1_, PM_2.5_, PM_10_, RH, temperature, and ultrafine particle count was examined. On this day (May 19), the lightning occurs (e.g.3 strikes) from nearly 11:45 AM to 13:00 PM local time with intensity more than 20 MeV is shown in Figure 4a. The various air quality parameters, such as intensity, PM_1_, PM_2.5_, PM_10_, RH, atmospheric temperature (T), and particle density from the same day are plotted in Figure 4b. The values of PM_1_, PM_2.5_, and PM_10_ increased after the lightning strikes. During the lightning events, the air temperature appears to increase to a higher value (39°C), and then after the events, the temperature decreases. Similarly, the plot shows that the RH was found to decrease whenever the lightning strikes occurred, and the atmospheric temperature was high, indicating that RH is inversely proportional to the air temperature (as shown in this Figure 4b. Moreover, after the lightning strikes, the particles per cubic centimeter were also found to be higher (as shown in the bottom panel of Figure 4b), indicating that lightning plays an important role in increasing the particle density in the atmosphere. As mentioned by Jnanesh et al. [13], there is a critical role of RH in regulating variations of lightning activity. Their study also observed that larger cloud droplets are associated with higher RH, whereas smaller droplets correspond to lower humidity levels. Atmospheric humidity primarily determines whether aerosols have a positive or negative impact on lightning. A greater lightning flash density with AOD is associated with lower RH, whereas higher humidity shows the opposite trend. This suggests that atmospheric humidity influences how aerosols affect lightning activity.

The analysis revealed a positive correlation between lightning intensity and air temperature, as temperatures increased during lightning events. Conversely, RH showed a negative correlation, as decreasing RH causes the increase in lightning intensity. Additionally, PM (PM_1_, PM_2.5_, and PM_10_) concentrations followed an upward trend, peaking alongside lightning intensity. These changes were recorded between 11:45 and 13:15, spanning approximately an hour. The findings, depicted in Figure 4b, emphasize the impact of lightning activity on atmospheric conditions and air quality during the premonsoon season. Similarly, Figure 5a also represents the lightning events that occurred on June 5in the premonsoon period as no monsoon began in early June. The Figure 5b represents the diurnal variations of air quality parameters in the corresponding day. The values of aerosol particulates PM_1_, PM_2.5_ and PM_10_ increase after the lightning strikes. During the lightning strikes (14:50–15:10 PM), as represented by peak intensity, the air temperature is found to be higher (about 33°C) and thereafter the temperature gradually decreases. Similarly, the RH is found to have decreased during the lightning events, and the atmospheric temperature is high, indicating that RH is inversely proportional to the air temperature (as shown in this Figure 5b). Moreover, after the lightning strikes, the ozone particles per cubic centimeter are also found to be higher (bottom panel of Figure 5b), indicating that lightning plays an important role in increasing the ozone particle density in the atmosphere.

Similar to observations from May 19, the temperature was found to be increased during the lightning events, and RH increased immediately after the lightning event. Other air quality parameters, including PM_1,_ PM_2.5_, PM_10_, and ultrafine particle count, showed an increasing trend, following a pattern similar to that observed in Figure 4. These findings, depicted in Figure 5, indicate that while lightning influences air quality parameters, its effects on temperature and humidity may vary depending on atmospheric conditions during the premonsoon season. As mentioned earlier, during the monsoon period, 2 days of lightning events (July 6 and 16, 2024) were analyzed. The atmospheric pollutants PM_1_, PM_2.5_, and PM_10_ increased, as shown in Figure 6b, after the occurrence of lightning events. Similarly, during strong lightning events, PM_1_ concentrations increased by an average of 12%, PM_2.5_ by ~ 18%, PM_10_ by ~ 12%, and ozone by ~ 22% compared with prelightning background levels. Ambient temperature also showed a rise of ~ 1.5°C–2.0°C in the immediate period of strong lightning strikes. These findings provide clear evidence of the magnitude of pollutant and thermal changes associated with lightning events. As mentioned earlier in several studies during the lightning phenomena, the atmospheric temperature is about five times the temperature of the surface of the Sun, which is about 30,000°C [5]. It is high enough, which is also shown from the plots (Figure 6b), and then, after the lightning, the temperature gradually decreases. Similarly, results show that the RH is inversely proportional to the temperature.

On July 6, 2024, during the monsoon period, lightning activity was recorded between 00:30 PM and 02:30 PM. Consistent with previous monsoon observations, temperature increased immediately after the lightning event, whereas RH decreased. However, unlike in the premonsoon period, air quality parameters PM_1_, PM_2.5_, and PM_10_ and ultrafine particle count followed a different pattern. Instead of increasing, their concentrations declined as lightning activity intensified. These findings, illustrated in Figure 6b, suggest that the effects of lightning on air quality parameters vary between the premonsoon and monsoon seasons, likely due to differences in atmospheric dynamics and precipitation.

Similarly, on July 16, 2024, during the monsoon period, lightning activity was recorded between 15:00 PM and 16:00 PM as shown in Figure 7b top panel as the peak values of intensity. Unlike the monsoon observation on July 6, temperature decreased, whereas RH increased as lightning intensity became peak. However, similar to the July 6 event, air quality parameters including PM_2.5_, PM_10_, PM_1_, and ultrafine particle count declined with increasing lightning activity. This pattern contrasts with the premonsoon period, where air quality parameters exhibited an upward trend during lightning events. These findings highlight the significant role of seasonal variations in shaping the atmospheric response to lightning activity. As mentioned by Li et al. [47], the lightning activity is also affected by aerosol effects which are mainly regulated by humidity and the relation between aerosols and lightning is opposite in dry cases to wet cases. Similarly, the urbanization and air pollution in cities affect lightning activity [48]. Khain et al. [32] used a cloud model that includes detailed microphysics, CNN, and ice nuclei, showing that aerosol‐rich clouds lead to stronger convection and more developed thunderstorms. Polluted urban areas often experience higher lightning activity [15, 49].

Aerosol particles are known to influence lightning, as regional studies have found a positive correlation between AOD and lightning density [50]. Lightning activity generally increases on days with higher AOD compared with days without lightning [51]. At high aerosol concentrations, their radiative impact can become dominant, suppressing convection and consequently decreasing lightning activity [36, 52]. The intricate interaction between aerosol microphysical and radiative effects, along with environmental and thermodynamic factors, plays a crucial role in shaping convection, electrification, and lightning activity. Hence, the lightning changes the atmospheric parameters and plays a very important role in the atmospheric environment.

Moreover, lightning generates high‐temperature plasma channels that dissociate molecular nitrogen and oxygen, producing reactive nitrogen oxides (NO and NO_2_), which subsequently contribute to ozone formation through photochemical reactions. Thunderstorm‐associated updrafts and turbulence further facilitate the vertical transport of surface pollutants into the mid‐troposphere, thereby enhancing localized pollutant concentrations in the vicinity of lightning events. The rapid heating also promotes secondary aerosol formation, which explains the observed increase in PM during lightning activity. While previous studies have largely examined lightning activity in relation to meteorological variables such as rainfall, RH, and temperature, our work extends this scope by integrating high‐resolution ground‐based lightning electric field data with localized pollutant measurements in the Kathmandu Valley. The analysis provides direct evidence of ozone and pollutant enhancement coinciding with lightning strikes and establishes a link between urban air pollution dynamics and lightning occurrence in a region where such investigations remain limited. To our knowledge, this represents the first integrated study of its kind in the cup‐shaped Himalayan basin of Kathmandu. The findings hold significant implications for air‐quality management, particularly in refining forecasting models during monsoon and premonsoon thunderstorms; for public health, by highlighting potential short‐term exacerbations of respiratory and cardiovascular conditions due to pollutant spikes; and for climate studies, by contributing to the broader understanding of lightning–pollution interactions and their role in atmospheric chemistry and radiative forcing in polluted high‐altitude environments.

## 4. Conclusion

In order to record lightning events, the electric field mill (EFM‐100) short‐range sensor has been installed at Kathmandu Bernhardt College, Bafal, Kathmandu. The lightning strikes recorded were within a radial distance of approximately 38 km from the instrument deployment center in Kathmandu. It constantly monitors lightning activity and displays alerts on a computer along with a real‐time graph of the electrical field in the atmosphere and the distance of striking. The lightning strikes were recorded by the EFM‐100 deployed at the measuring station, Kathmandu over a 57‐day period between May 19 and August 17, 2024, covering both the premonsoon and monsoon seasons. On some days, data were missing due to problems with electric power supply and technical problems with the instruments. The observations reveal that lightning strikes occurred almost every day as presented in Table 1.

The impact of lightning on atmospheric pollutants such as PM_1_, PM_2.5_, PM_10_, RH, temperature, and ozone particle density (per cubic centimeter) was analyzed. The results clearly show that the atmospheric pollutants PM_1_, PM_2.5_, and PM_10_, found to be higher during the lightning events. PM_2.5_ concentrations increased by an average of approximately 18%, PM_10_ by about 12%, and ozone by about 22% compared with prelightning background levels. Similarly, the atmospheric temperature tends to be high during lightning events, having increased by 1.5°C–2.0°C during the strong lightning events followed by a gradual decrease thereafter. It was also observed that RH is inversely proportional to temperature, as it rises during lightning events. This clearly demonstrates that RH and temperature are inversely related. Furthermore, it can also be inferred that lightning plays an important role in increasing the concentration of ozone particles in the atmosphere, as the density of ozone particles per cubic centimeter increases following a lightning event. These findings indicate that lightning events have a significant impact on atmospheric air quality parameters. This study not only provides insights into air pollution–lightning interactions in the Himalayan region, like Nepal but also contributes to the global understanding of how anthropogenic pollutants can influence atmospheric electricity. This finding also highlights potential applications in climate research, urban air quality management, and disaster preparedness, underscoring the global significance of this study.

## Conflicts of Interest

The authors declare no conflicts of interest.

## Author Contributions

Dr. P. B. A.: conceptualization, data curation, formal analysis, funding acquisition, methodology, software, validation, writing—original draft, data visualization. Mr. J. S. D.: data curation, formal analysis, methodology, software, data visualization. Prof. K. N. P.: funding acquisition, resources, supervision, writing—review and editing, data visualization. Prof. N. P. C.: conceptualization, funding acquisition, project administration, resources, supervision, writing—review and editing, data visualization.

## Funding

This study was supported by the Research Coordination and Development Council (RCDC), TU, Nepal (NPAR‐079/80‐ERG‐04).

## Data Availability

The data that support the findings of this study are available on request from the corresponding author. The data are not publicly available due to privacy or ethical restrictions.
